# Efficacy of social cognition and interaction training in outpatients with schizophrenia spectrum disorders: randomized controlled trial

**DOI:** 10.3389/fpsyt.2023.1217735

**Published:** 2023-08-04

**Authors:** Joanna M. Fiszdon, H. Drew Dixon, Charlie A. Davidson, David L. Roberts, David L. Penn, Morris D. Bell

**Affiliations:** ^1^VA Connecticut Healthcare System, West Haven, CT, United States; ^2^Department of Psychiatry, School of Medicine, Yale University, New Haven, CT, United States; ^3^Department of Psychology, College of Health Professions, Mercer University, Macon, GA, United States; ^4^Department of Psychology, Emory University, Atlanta, GA, United States; ^5^Akin Mental Health, San Francisco, CA, United States; ^6^Department of Psychiatry, University of Texas Health Science Center, San Antonio, TX, United States; ^7^Department of Psychology and Neuroscience, The University of North Carolina at Chapel Hill, Chapel Hill, NC, United States

**Keywords:** social cognition, randomized controlled trial, schizophrenia, psychosis, training, rehabilitation

## Abstract

Given the relationship between social cognition and functional outcome in schizophrenia, a number of social cognitive interventions have been developed, including Social Cognition Interaction Training (SCIT), a group-based, comprehensive, manualized intervention. In the current trial, we examined SCIT efficacy as well as potential moderators of treatment effects. Fifty-one outpatients were randomized to SCIT or a wait-list-control (WLC), with assessments of social cognition, neurocognition, self-report, symptoms, and functioning conducted at baseline and end of the active phase. Relative to WLC, we did not find significant improvements for SCIT on neurocognition, social cognition, self-report, or symptoms, though there was a trend-level, medium effect favoring the SCIT condition on interpersonal and instrumental role function. Post-hoc analyses indicated that baseline neurocognition did not impact degree of social cognitive or functional change. Shorter duration of illness was significantly associated with better post-training neurocognition and self-esteem and, at trend-level with better symptoms and social functioning. We discuss the importance of outcome measure selection and the need for continued evaluation of potential treatment moderators in order to better match people to existing treatments.

**Clinical trial registration**: Clinicaltrials.gov, Identifier NCT00587561.

## 1. Introduction

Cognitive impairments found in schizophrenia-spectrum disorders (SSD) can be further specified as deficits in neurocognition, which includes abilities such as attention, memory, and executive function, and deficits in social cognition (1), which refers to “the ability of individuals to understand themselves and others in the wider context of social interactions, especially others’ thoughts, feelings, and intentions” [(2) p. 80]. Impairments in social cognition have been shown to predict poorer social functioning (1, 3, 4), as well as to mediate the link between neurocognition and functional outcomes in people with schizophrenia spectrum disorders (SSD) (5–7).

Given the relationship between social cognition and functional outcome, a number of social cognitive interventions have been developed, ranging from single-domain, targeted treatments, to comprehensive treatments that aim to improve multiple domains of social cognition (8, 9). In one of the first meta-analyses of social cognitive treatment trial data, these varied approaches to social cognitive treatments have collectively been associated with moderate-to-large effects on emotion perception, psychiatric symptoms, and observer-rated functioning, with small-to-moderate effects on Theory of Mind (ToM), and no significant effects on attributional style (10).

Among comprehensive social cognitive interventions, probably the best known and most widely studied has been Social Cognition and Interaction Training (SCIT). This manualized group intervention was developed based on empirically-derived models of social cognition in schizophrenia. While SCIT can be subsumed within cognitive behavior therapy (CBT), it is distinguished from it by an emphasis on the process of how patients reach erroneous conclusions, rather than the fallacy of the conclusions themselves. Social cognitive domains specifically targeted by SCIT include emotion perception, attributional biases, and ToM (11). Initial feasibility of SCIT was first demonstrated in 2005 (12), followed by a number of small trials again supporting feasibility and suggesting preliminary efficacy (13–16). SCIT has also been implemented in a variety of treatment settings, across different phases of illness and clinical populations [e.g., (17–19)], and across different cultures (20–22). A 2016 critical review and meta-analysis of controlled comprehensive social cognitive training interventions (2), which included four RCTs and two quasi-experimental trials of SCIT, reported large effects for emotion perception, moderate effects for ToM, small to medium effects for attributional style, and small effects for symptoms. While these results were promising, the total number of RCTs examined was small, the review included different social cognitive treatment approaches and did not perform subgroup analyses specifically for SCIT, and the authors cautioned that more attention was needed to assure treatment fidelity and unbiased (i.e., blinded) outcome ratings. Nevertheless, each of the six trials of SCIT included in the review reported significant benefits for SCIT in social cognition, symptoms, and/or functioning.

A handful of additional RCTs of SCIT for SSD have been published more recently. While several of these provide strong data in support of SCIT [e.g., (23)], a number of reports are less compelling with conclusions about efficacy based on within-group and not between group differences (24), analyses that only included those who completed a certain proportion of training sessions (25), small sample sizes with significant drop-out (17) and small to large effects that did not reach statistical significance (21). Some investigators have also reported failure to find significant group differences on any measures (26–28). While two of these trials (27, 28) included active control conditions consisting of either cognitive remediation or targeted training in affect recognition, which may reasonably be thought to impact neurocognition or affect recognition narrowly, neither trial found group differences on measures of abilities uniquely targeted by SCIT. Another trial comparing SCIT to a therapeutic alliance group and treatment as usual (20) reported limited and somewhat mixed effects on measures of social cognition and social functioning and a relationship between therapeutic alliance and improvement. The authors interpreted improvements in measures of interest found in both active conditions as due to shared therapeutic factors, which could potentially also explain the lack of group differences in the other two trials with active controls. It is also worth noting that many of the trials did not include fidelity ratings and that several of the above trials were international and included adaptations to SCIT, which may have resulted in qualitatively different interventions than that originally designed for the United States. Given these equivocal findings and what continues to be a relatively small literature base, additional data is needed on the efficacy of SCIT.

In the current randomized controlled trial (RCT), we sought to evaluate the efficacy of social cognition and interaction training (SCIT). Outpatients diagnosed with schizophrenia-spectrum disorders and engaged in treatment were randomized into one of two conditions: (1) a 20 to 24 session manualized SCIT group administered weekly over the course of approximately 6 months, or (2) wait-list control (WLC). Social cognition, basic cognition, symptom and community function data were collected at pre-and post. Our primary question focused on the impact of SCIT on social cognitive function. We hypothesized that relative to WLC, SCIT would lead to significantly greater improvement on measures indexing affect perception, theory of mind, and attributional bias. Our secondary question focused on the impact of SCIT on neurocognition, symptoms, and functioning, and again we hypothesized that SCIT would be associated with improvements in these areas. Based on prior data indicating heterogeneity in SCIT effects, we also performed post-hoc analyses examining the potential impact of neurocognition, session attendance, and duration of illness on SCIT efficacy.

## 2. Methods

### 2.1. Participants

Participants were recruited *via* flyers, word-of-mouth and presentations to treatment teams at a VA medical center and a community mental health clinic. In order to be eligible, participants had to meet the following criteria: Diagnostic and Statistical Manual (DSM-IV-TR) (29) diagnosis of schizophrenia or schizoaffective disorder, age 18–65, psychiatric stability (at least 30 days since last psychiatric hospitalization or change in psychiatric medications), currently receiving outpatient mental health services, no housing changes in past 30 days, not meeting criteria for substance use disorder in past 30 days, no significant head trauma, and no auditory or visual impairment that would interfere with study procedures. The study was approved by local Institutional Review Boards, and all participants provided written informed consent.

### 2.2. Measures

In addition to collecting baseline demographic, diagnostic (Structured Clinical Interview for DSM-IV, SCID, (30)), premorbid intelligence (Wide Range Achievement Test 3, Reading, WRAT-3 Reading, (31)) and current intelligence (Wechsler Abbreviated Scale of Intelligence, WASI, (32)) information, pre-post active phase data was collected on measures of social cognition, neurocognition, psychiatric symptoms, and functioning.

#### 2.2.1. Social cognition

Emotion perception, Theory of Mind (ToM) and attributional style were assessed. The Facial Emotion Identification Test FEIT; (33) was used to assess emotion perception. Examinees are presented with 19 photographs of faces expressing one of six basic emotions (happy, sad, angry, afraid, surprised, and ashamed), and asked to choose which emotion is portrayed. The Hinting Task (34, 35) was used to assess ToM. Examinees are presented with 10 vignettes of dyad social interactions and asked to make inferences about the intent behind a hint dropped by one of the characters. Scores for each vignette range from 0–2, based on whether the examinee is able to identify the intent without (2) or with (1) prompts from the examiner. The Internal, Personal and Situational Attributions Questionnaire (IPSAQ, (36)) was used to assess attributional style. Stimuli consist of 16 positive and 16 negative social situations. The examinee is asked to provide an explanation for why they think a certain outcome occurred (e.g., a friend betrayed the trust you had in her), and indicate whether what they thought caused the outcome had to do with something about the examinee (internal), something about the other person described in the vignette (personal), or something about the situation itself (situational). Two cognitive bias scores are generated. An Externalizing Bias (EB) index is computed by subtracting the number of internal attributions for negative events from the number of internal attributions for positive events, with positive index scores reflecting a self-serving bias, namely being more likely to attribute oneself as the cause of positive events than of negative events. The Personalizing Bias (PB) index is computed by dividing the total number of personal attributions by the sum of both personal and situational attributions for negative events, with index scores over 0.5 reflecting the tendency to be more likely to attribute the cause of negative events to other people rather than situational factors. Emotional intelligence was assessed using the social cognitive domain from the Matrics Consensus Cognitive Battery (MCCB, (37)), namely the Mayer-Salovey-Caruso Emotional Intelligence Test (MSCEIT), Emotional Intelligence subtest.

#### 2.2.2. Neurocognition

The MCCB was used to index neurocognitive function. The MCCB consists of 10 tests that provide a comprehensive assessment of 7 cognitive domains. The MCCB was developed by an expert panel of researchers, under a National Institutes of Mental Health (NIMH) contract, as a broad yet sensitive measure to assess cognitive change in treatment studies. The battery includes alternate test forms for repeated administrations. A composite score based on 6 of the 7 domain scores was created (the social cognitive domain was omitted from the 6-domain composite).

#### 2.2.3. Symptoms

The Positive and Negative Syndrome Scale (PANSS, (38)) is a well-known interviewer-rated scale indexing the core symptoms of schizophrenia as well as a broad range of general psychiatric symptoms. Each symptom is rated on a Likert-type scale ranging from 1–7, for total score range of 30 to 120. In addition to total score, we also used Bell and colleague’s 5-factor solution, consisting of positive, negative, cognitive, hostility, and emotional distress factors (39). Self-report measures included the Brief Fear of Negative Evaluations-II scale (BFNE-II; (40)), the Social Anxiety and Distress Scale (SADS; (41)), the Rosenberg Self Esteem Scale (42), and the Beck Cognitive Insight Scale (BCIS, (43)). The BCIS consists of a subscale assessing self-reflectiveness (SR), or the ability to reflect on and question one’s own conclusions, and self-confidence (SC), or overconfidence about one’s interpretations of events and receptivity to feedback.

#### 2.2.4. Functioning

Current functioning was assessed using performance-based, self-report and interviewer-rated measures. The Independent Living Scales Survey (ILSS, (44)) is a measure of community function that includes both self-report and examiner-rated items on: Appearance and Clothing, Personal Hygiene, Food Preparation, Health Maintenance, Money Management, and Leisure Activities. For each subdomain, a score between 0 and 1 is computed, and total score is the average of subdomain scores. The Quality of Life Scale (QLS; (45)) is an interviewer-rated measure of the respondent’s social, occupational, and interpersonal functioning. In addition to total score, scores in four domains are computed: interpersonal functioning, intrapsychic foundations, instrumental role functioning, and common objects and activities. The Social Functioning Scale (SFS, (46)) is an informant-rated (in this case rated by research staff with input from participants) measure of community function. It distinguishes lack of competence from lack of performance of basic skills and behaviors, which is a particularly important distinction for schizophrenia research. In addition to total score, seven subdomain scores are computed. The Social Skills Performance Assessment (SSPA, (47)) is a role-play proxy measure of conversational skills with two sets of conversations focused around meeting a new neighbor and negotiating with a landlord to fix something. All SSPA ratings were done by the same assessor, who was not blind to treatment condition.

### 2.3. Procedures

Randomization to SCIT or WLC was stratified based on social cognitive function (cut score of 11 or more on the FEIT). Separate randomization schedules were used within each of the 2 stratum using block randomization (blocks of 12) to assure similar sample sizes across conditions. Randomization was performed by statistical assistant not otherwise associated with the study. Assessments occurred at baseline and end of the 6-month active SCIT phase. Doctoral-level research staff were trained on all study measures by first author (JMF), and inter-rater reliability (intraclass correlation coefficient) was >0.80 for symptom ratings. Not all assessors were blind to condition. All SCIT groups were co-led by two doctoral-level staff who had been trained on this manual-based intervention. First author JMF, who received training on the SCIT intervention from its developers served as one of the SCIT group facilitators for each cohort. Participants were paid for assessments. As this was an efficacy and not an effectiveness evaluation, those randomized to SCIT were also paid for attending SCIT sessions. Throughout the study period, all participants continued to receive their standard mental health treatment consisting of case management, individual or group psychotherapy, other psychosocial interventions such as vocational rehabilitation, and/or medication management, as warranted and as requested.

#### 2.3.1. SCIT group

SCIT is a manualized, comprehensive social cognitive intervention which was delivered over 20–24 1-h, weekly groups and led by two facilitators. SCIT consists of three content phases: Emotion Training, which focuses on establishing the relationship between feelings, thoughts and situations, identifying basic emotions, using behavioral cues in emotion perception, and discussing suspiciousness as one of the basic emotions; Figuring Out Situations, which focuses on not jumping to conclusions, identifying attributional biases, tolerating ambiguity, distinguishing facts from guesses, and gathering data; and Integration/Checking It Out, where the focus is on consolidating skills, applying what was learned to participants’ lives, and learning how to appropriately check out assumptions. Over the course of the training, the focus of SCIT changes from “cold” cognition, interpreting the social interactions of others, to “hot” cognition, interpreting social interactions pertaining to oneself. Client motivation and engagement are seen as particularly important and are enhanced through the use of shaping, Socratic questioning, ecologically valid video-clips, role-plays, engaging games, and suggested homework assignments.

### 2.4. Power analysis

As a guide to statistical power in this study, we considered pilot data from a SCIT feasibility study (12), which provided FEIT scores pre-and post-treatment. In this study, pre-treatment FEIT values were 11.3 ± 2.1 while post-treatment levels were 13.5 ± 2.6. We assumed little change in FEIT scores for the WLC condition. Based on a repeated measures design with an overall SD = 2.6, alpha = 0.05, n = 24 subjects per group, and pre-post correlation = 0.5, assuming pre-and post-FEIT scores of 11.3 for WLC and 11.3 and 13.5, respectively for SCIT, we calculated we would have >90% power to detect such an interaction effect (ES = 0.52) between treatment and time.

### 2.5. Data analysis

Repeated measures analyses of variance (RMANOVAs) with Group (SCIT vs. WLC) x Time (pre-intervention vs. post-intervention) interactions were conducted to examine the impact of SCIT on measures of social cognition, neurocognition, symptoms and functioning. All available data was used for these analyses. While the number of comparisons was large, we chose to retain the standard *p* < 0.05 significance level so as not to overlook potential improvements in what is admittedly a small sample size. This approach is consistent with many prior SCIT evaluations. We additionally calculated effect size estimates for between-group comparisons using partial eta squared, with 0.01 representing a small effect, 0.06 representing a medium effect, and 0.14 representing a large effect. In order to examine potential moderating effects of session attendance, duration of illness, and baseline neurocognition on SCIT efficacy, as additional post-hoc analyses we also computed partial correlations between these three variables and post-training measures of social cognition, neurocognition, self-report, symptoms, and functioning, controlling for baseline values. These post-hoc analyses were conducted for those randomized to SCIT condition.

## 3. Results

**Figure 1:**
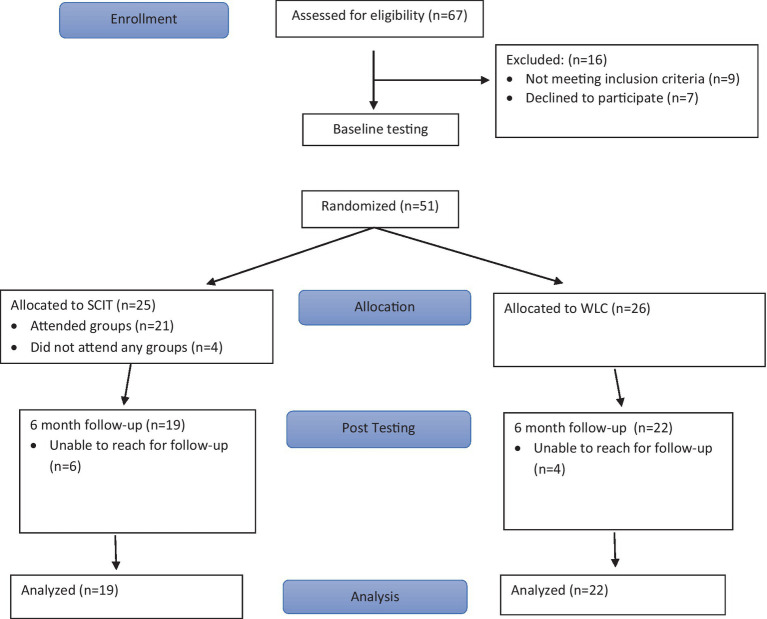
CONSORT flow diagram.

Please refer to Figure 1 for CONSORT flow chart. Of 67 volunteers assessed for eligibility, fifty-one completed baseline testing and were randomized to SCIT (*n* = 25) or Waitlist Control (WLC, *n* = 26). Four participants randomized to SCIT did not attend any group sessions. Six-month follow-up (at end of the 6-month SCIT active phase) data was obtained for 22 of the participants randomized to WLC (85%), and for 19 of the participants randomized to SCIT (76%), which does not represent a significant difference in attrition rates. Of the 25 participants randomized to SCIT, including 4 that did not attend any SCIT groups, average attendance was 53%. For those who attended at least one SCIT session, average attendance was 63%. Approximately half (52%) of those randomized to SCIT attended 75% or more of the sessions. There were no significant demographic differences between treatment conditions at baseline assessment (see Table 1), though it should be noted that this was an older sample with an average age of approximately 48, and with 41% of the sample aged 50 or older. At baseline there were also no significant differences on demographic nor any other measures between those who completed the 6-month assessments versus the 11 participants who were lost to follow-up. No adverse effect or negative outcomes related to study procedures were noted for either condition.

**Table 1:** Baseline demographics of participants randomized to SCIT and Waitlist Control conditions.

Variables	SCIT (*n* = 25) Mean (SD)	WLC (*n* = 26) Mean (SD)
Age	45.56 (10.97)	49.96 (9.15)
Education (years)	12.00 (2.45)	12.31 (2.28)
Gender (% male)	60%	62%
Race (% white)	28%	39%
Marital status (% never married)	56%	46%
WASI IQ	87.12 (14.33)	88.88 (14.76)
Age of onset	19.13 (6.90)	22.73 (8.35)
Schizophrenia (%)	88%	81%
No. Hospitalizations	9.36 (10.78)	11.48 (19.06)
GAF	40.52 (8.05)	41.15 (7.35)
MCCB t-score	28.46 (13.91)	27.27 (11.57)
PANSS	55.50 (15.05)	56.38 (17.62)

* *p* < 0.05; no significant differences were found between groups on any variables.

WASI IQ, Wechsler Abbreviated Scale of Intelligence, Intelligence Quotient; GAF, Global Assessment of Functioning Scale; MCCB, Matrix Consensus Cognitive Battery; PANSS, Positive and Negative Syndrome Scale.

Results of the repeated measures analyses are summarized in Table 2. There was no significant group x time interaction for neurocognition. For social cognition, the only significant group x time interaction was found for the FEIT, however this appeared to be due to the WLC group deteriorating in emotion recognition performance at six-month assessment, while scores remained stable in the SCIT group. To further underscore this point, the within-subjects effect size using Cohen’s d was 0.65 for the WLC condition, with estimated power at 83% given post-training sample size. For the SCIT condition, the within-subjects effect size was 0.05, with power of only 5.5% given post-training sample size. For symptoms, there was a significant interaction for PANSS total and the PANSS Cognitive factor (with a trend level change for PANSS Hostility factor) however these appeared mostly due to the WLC improving at 6 months. There were no significant group x time interactions on self-report measures of self-esteem, fear of negative evaluations, social anxiety, or cognitive insight. There were no significant group x time interactions for the SFS, ILSS, or SSPA, however there was a trend-level (*p* < 0.10), medium effect for QLS Interpersonal and Instrumental Role Function subscales, this time with the SCIT condition showing improvement.

**Table 2:** Outcome measures by treatment group.

Variable	SCIT		WLC			Group x Time interaction
	Pre *M* (SD)	Post *M* (SD)	Pre *M* (SD)	Post *M* (SD)	*F*	ηp2
Social cognition						
FEIT	10.16 (2.83)	10.26 (2.78)	10.86 (2.30)	9.18 (3.02)*	6.03*	0.13
Hinting	15.95 (2.93)	16.58 (2.79)	15.86 (3.44)	17.41 (2.55)	0.99	0.03
IPSAQ EB	3.47 (3.20)	3.74 (2.77)	2.55 (3.64)	2.27 (3.84)	0.21	0.01
IPSAQ PB	0.54 (0.29)	0.59 (0.36)	0.65 (,27)	0.60 (0.27)	0.86	0.02
MSCEIT Emotional intelligence T score	38.11 (8.60)	36.21 (6.77)	31.73 (11.21)	34.18 (11.38)	2.62	0.06
Neurocognition						
MCCB 6-scale composite T score	36.07 (10.38)	37.49 (10.74)	37.74 (7.68)	37.67 (7.48)	1.17	0.03
Symptoms						
PANSS Total	55.50 (15.05)	55.75 (14.29)	56.38 (17.62)	48.71 (9.44)	4.90*	0.12
Positive	13.39 (4.39)	12.44 (5.40)	13.10 (6.62)	9.81 (4.04)	2.32	0.06
Negative	13.00 (5.40)	12.58 (4.05)	13.33 (4.78)	12.67 (3.88)	0.02	0.00
Cognitive	12.06 (4.13)	13.35 (3.87)	13.05 (5.02)	11.62 (3.34)	6.52*	0.15
Emotional	8.63 (3.62)	8.79 (4.38)	9.62 (4.36)	8.52 (3.56)	1.68	0.04
Hostility	5.94 (2.58)	5.89 (2.35)	6.05 (2.65)	4.86 (1.59)	**3.16 #**	0.08
RSES total	28.74 (5.55)	29.68 (6.84)	26.86 (6.00)	30.00 (5.80)	1.82	0.05
BFNE-II total	15.32 (11.32)	11.56 (8.48)	13.86 (9.59)	11.14 (7.85)	0.04	0.00
SADS total	11.63 (7.32)	11.89 (8.18)	16.91 (7.32)	14.27 (8.84)	2.24	0.05
BCIS-SR	14.68 (6.30)	14.79 (6.86)	11.82 (4.58)	14.18 (5.60)	1.60	0.04
BCIS-SC	9.11 (4.47)	9.11 (4.77)	8.82 (3.92)	9.00 (4.86)	0.02	0.00
Functioning						
SFS total	755.65 (55.51)	781.36 (53.63)	757.14 (36.11)	767.42 (45.77)	0.36	0.01
Social engagement	104.94 (12.24)	108.26 (10.39)	103.85 (11.98)	105.9 (13.12)	0.07	0.00
Interpersonal communication	120.42 (20.27)	124.11 (14.47)	115.67 (13.5)	118 (16.91)	0.03	0.00
Independence (p)	106 (15.87)	112.44 (11.39)	111.26 (9.8)	113.09 (10.65)	0.80	0.02
Independence (c)	111.71 (11.71)	113.31 (13.2)	111.93 (10.1)	112.84 (11.2)	0.12	0.00
Employment	99.5 (13.54)	101.34 (12.32)	105.16 (11.23)	104.47 (12.69)	0.21	0.01
Recreation	107.31 (12.06)	108.94 (15.01)	102.26 (9.43)	106.85 (15.55)	0.22	0.00
Prosocial activities	105.76 (15.97)	112.94 (12.71)	106.76 (10.47)	106.83 (10.48)	2.14	0.05
QLS Total	65.21 (17.89)	76.21 (21.65)	68.67 (18.59)	72.38 (18.07)	2.43	0.06
Interpersonal	26.37 (9.01)	30.47 (8.41)	26.90 (8.50)	26.52 (8.34)	**3.09 #**	0.08
Intrapsychic	28.95 (7.16)	29.68 (6.64)	28.29 (5.49)	31.05 (4.47)	1.41	0.04
Objects and Activities	7.58 (2.63)	8.00 (1.73)	8.14 (2.24)	7.95 (1.56)	1.20	0.03
Instrumental Role	2.32 (5.50)	8.05 (8.94)	5.33 (7.18)	6.86 (7.44)	**3.42 #**	0.08
SSPA	4.10 (0.63)	4.22 (0.48)	4.03 (0.55)	4.04 (0.68)	0.47	0.01
ILSS	0.74 (0.15)	0.75 (0.15)	0.74 (0.10)	0.76 (0.12)	0.20	0.01

# *p* < 0.10, * *p* < 0.05 (significant Group x Time interaction, RMANOVA).

FEIT, Facial Emotion Identification Task; IPSAQ, The Internal, Personal, and Situational Attributions Questionnaire (EB, externalizing bias; PB, personalizing bias); MSCEIT, Mayer-Salovey-Caruso Emotional Intelligence Test; PANSS, Positive and Negative Syndrome Scale; RSES, Rosenberg Self Esteem Scale; BFNE, Brief Fear of Negative Evaluations Scale; SADS, Social Avoidance and Distress Scale; BCIS, Beck Cognitive Insight Scale (SR, self-reflection; SC, self-confidence); SFS, Social Functioning Scale; ILSS, Independent Living Skills Survey; QLS, Quality of Life Scale; SSPA, Social Skills Performance Assessment; ηp2, partial eta-squared.

For the SCIT condition, partial correlations between sessions attended, duration of illness, and neurocognition with post-training measures of social cognition, neurocognition, self-report, symptoms, and functioning and controlling for their baseline values, are reported in Table 3. Session attendance correlated negatively at trend level with PANSS Hostility but no other outcome measures. There were significant negative correlations between duration of illness and neurocognition (*r* = −0.596, *p* = 0.015) as well as self-esteem (*r* = −0.520, *p* = 0.032) at post-treatment. There was also a trend-level positive correlation between duration of illness and PANSS total at post-treatment (*r* = 0.511, *p* = 0.062), as well as a trend-level negative correlation between duration of illness and SFS total (*r* = −0.474, *p* = 0.054) at end of treatment, in both cases with lower duration of illness associated with better outcomes.

**Table 3:** Partial correlations between percent of sessions attended, duration of illness, and neurocognition with post-training performance, controlling for baseline performance (SCIT condition only).

Variable	Partial correlations		
	% sessions attended	Duration of illness	MCCB composite
Social cognition			
FEIT	0.171	0.268	−0.164
Hinting	0.220	0.087	−0.096
IPSAQ EB	0.278	−0.063	0.144
IPSAQ PB	−0.059	0.011	0.394
MSCEIT ME T	−0.157	−0.038	−0.014
Neurocognition			
MCCB 6-scale composite T	−0.310	−0.596 *	n/a
Symptoms			
PANSS Total	−0.065	**0.511 #**	0.160
Positive	0.291	0.361	0.325
Negative	−0.324	0.022	−0.140
Cognitive	0.294	0.318	0.167
Emotional	0.245	0.402	0.082
Hostility	−0.453	0.391	**−0.428 #**
RSES total	−0.206	−0.520 *	0.163
BFNE-II total	−0.126	0.128	−0.233
SADS total	0.086	−0.042	0.154
BCIS-SR	−0.284	−0.339	0.069
BCIS-SC	0.119	0.232	−0.282
Functioning			
SFS total (avg)	0.251	**−0.474 #**	0.183
QLS Total	0.117	−0.145	−0.040
Interpersonal	0.117	−0.145	−0.040
Intrapsychic	0.134	−0.294	0.002
Objects and Activities	0.038	0.339	0.268
Instrumental Role	0.081	−0.017	0.279
SSPA	0.311	0.290	0.001
ILSS	0.403	0.194	0.004

# *p* < 0.10, * *p* < 0.05.

FEIT, Facial Emotion Identification Task; IPSAQ, The Internal, Personal, and Situational Attributions Questionnaire (EB, externalizing bias, PB, personalizing bias); MSCEIT, Mayer-Salovey-Caruso Emotional Intelligence Test; PANSS, Positive and Negative Syndrome Scale; RSES, Rosenberg Self Esteem Scale; BFNE, Brief Fear of Negative Evaluations Scale; SADS, Social Avoidance and Distress Scale; BCIS, Beck Cognitive Insight Scale (SR, self-reflection; SC, self-confidence); SFS, Social Functioning Scale, ILSS, Independent Living Skills Survey; QLS, Quality of Life Scale; SSPA, Social Skills Performance Assessment.

## 4. Discussion

In the current RCT we evaluated the efficacy of SCIT, compared to WLC, on neurocognitive, social cognitive, self-report, symptom, and functional measures assessed at baseline and end of the 6-month active study period. Average attendance rate was 53%, increasing to 63% for those who attended at least one SCIT session, and with approximately half of the sample attending 75% or more of the sessions. Relative to WLC, we did not find significant improvements for SCIT on neurocognition, social cognition, symptoms, or self-report measures of self-esteem, fear of negative evaluations, social anxiety, or cognitive insight. From among the several measures of functioning that were administered, we found a trend-level (*p* < 0.10), medium effect favoring the SCIT condition on the QLS Interpersonal and Instrumental Role Function subscales.

In post-hoc analyses examining potential moderators of treatment effects in the SCIT condition, session attendance was not significantly correlated with social cognitive or functional improvements, though there was a trend level relationship between session attendance and PANSS Hostility, with lower post-training hostility in those with greater session attendance. Shorter duration of illness was significantly associated with better post-training neurocognition and self-esteem and, at trend level, with better symptoms and social functioning. Better baseline neurocognition did not impact social cognitive or functional improvements, though similarly to session attendance, better baseline neurocognition was associated at trend level with lower PANSS hostility scores.

Our findings regarding better outcomes in those with shorter illness duration are to some extent consistent with those of Kanie and colleagues (21), though in their case shorter duration of illness was associated with significant improvements in social cognition while in the current study none of the examined variables appeared to impact social cognition. Notably, Kurtz and colleagues (10) reported the opposite effect for duration of illness, with greater improvements in those with longer illness duration. This highlights the need to continue to examine potential moderators of treatment effects in order to learn what factors may impact efficacy and to both better match people to existing treatments as well as consider how treatments may need to be refined for some subgroups.

While recent meta-analyses (48, 49) suggest medium to large effects of social cognitive treatments on a range of variables, with particularly consistent evidence for measures of emotion perception and theory of mind, we failed to find significant effects of SCIT on the majority of our measures. Perhaps contributory to this were clinical characteristics of the sample including age and duration of illness. Our sample was older than typical for SCIT and many other treatment studies, with over 40% of the sample aged 50 or more. It is possible that older persons may not relate to SCIT content in the same way as younger individuals, that the type and quality of their social interactions differs, and/or that their social cognitive biases may be more entrenched than they are in younger individuals. As noted above, this deserves further study and may be an important factor to consider when offering SCIT groups.

Lack of effects could also be due to a low average attendance rate, only once weekly treatment intensity, or to other evidence-based clinical interventions that were available as part of treatment as usual. We also did not collect data on interactions with practice partners and rates of homework adherence. Especially in this older, more chronic population, more intensive instruction with plentiful opportunities for practice outside of the sessions may be necessary for skills taught during SCIT to evidence in measurable gains [e.g., (50)].

Although our findings do not provide support for the efficacy of SCIT, they are in line with several other recent RCTs. For example, in one of the largest RCT trials of SCIT, Dark and colleagues (26) failed to find any significant differences between SCIT and a befriending group control condition, even when analyses were repeated in a subsample of individuals who completed at least 50% of the training sessions. Lo and colleagues (25) also failed to find an advantage for SCIT over the control condition when data from all randomized participants was examined, and effects only emerged when they repeated the analyses with treatment completers, defined as those who attended ≥50% of the sessions. Gordon and colleagues (24) also failed to find group differences between SCIT and a waitlist control, though effects did emerge when additional data was included for those who received SCIT after a WLC phase, and within-group analyses were conducted.

The medium-sized effects favoring SCIT condition on QLS subscales of Interpersonal and Instrumental role function are in line with a number of other trials that also reported improvements on more distal measures of functioning [e.g., (23, 50, 51)], and bring to the forefront questions about what are the most important, “right” outcomes for this type of treatment—with real-world social function arguably a more meaningful outcome than changes on laboratory tasks assessing social cognition. In fact, some researchers have reported quite pronounced improvements on measures of real-world functioning in the absence of significant improvements on more proximal measures purportedly assessing domains being targeted by an intervention (52). Given however that our comparator was a waitlist control, we are not able to determine whether these social function changes represent improvements related to specific skills taught during SCIT or whether they represent non-specific treatment factors common to other forms of psychological interventions. Notably, Hasson-Ohayon and colleagues (20) reported that improvements in social cognition were related to therapeutic alliance, which was similar across the SCIT and another group comparator condition examined in their study.

The current study had a number of limitations. Not all assessors were blind to treatment condition and participants were aware of group allocation, though this would be more problematic and suggestive of potential bias if we had found strong effects favoring SCIT on interviewer-rated or self-report measures. Our sample was also relatively small, with an approximately 20% attrition at post-training. As has been suggested by many others [e.g., (53, 54)], it may have also been the case that the measures we used to index treatment effects were inappropriate—whether having poor psychometric properties broadly, not assessing the specific constructs or skills targeted by SCIT, or perhaps just not being sensitive enough to change. While only one of our social cognitive measures (Hinting Task) has been recommended by the large-scale social cognitive psychometric evaluation trial (55), the remaining measures are far from obscure and several other groups have found them sensitive to treatment effects [e.g., (10, 53)]. Other limitations include somewhat low treatment intensity (once weekly sessions), only fair treatment adherence, and lack of consistent fidelity monitoring.

Impairments in social function play a large role in a person’s level of disability, community integration, and likelihood of relapse, and as such need to continue to be a focus of treatment development and validation. While results of the current study are primarily negative, they nevertheless serve as important datapoints that, evaluated in aggregate with results of prior trials, may provide a more comprehensive, thorough picture of the efficacy of SCIT, likely active ingredients, and potential variables that impact treatment response.

## Data availability statement

The datasets presented in this article are not readily available because the full datasets contain identifying information, and data sharing is subject to facility guidelines. Requests to access the datasets should be directed to the corresponding author.

## Ethics statement

The studies involving human participants were reviewed and approved by VA Connecticut Human Studies Subcommittee. The patients/participants provided their written informed consent to participate in this study.

## Author contributions

JF and MB conceived of the study, with consultation from DR and DP. HD, CD, and JF conducted data analyses and drafted the manuscript. DR, DP, and MB made additional contributions to the manuscript. All authors contributed to the article and approved the submitted version.

## Funding

This study was funded by VA Rehabilitation Research and Development grant #D4628W to JF.

## Conflict of interest

The authors declare that the research was conducted in the absence of any commercial or financial relationships that could be construed as a potential conflict of interest.

## Publisher’s note

All claims expressed in this article are solely those of the authors and do not necessarily represent those of their affiliated organizations, or those of the publisher, the editors and the reviewers. Any product that may be evaluated in this article, or claim that may be made by its manufacturer, is not guaranteed or endorsed by the publisher.
